# Twelve weeks supplementation with an extended-release caffeine and ATP-enhancing supplement may improve body composition without affecting hematology in resistance-trained men

**DOI:** 10.1186/s12970-016-0136-9

**Published:** 2016-06-10

**Authors:** Jordan M. Joy, Roxanne M. Vogel, Jordan R. Moon, Paul H. Falcone, Matt M. Mosman, Michael P. Kim

**Affiliations:** Department of Nutrition and Food Sciences, Texas Woman’s University, 401 AME Drive #7101, Denton, TX 76207 USA; American Public University System, School of Health Sciences, Charles Town, WV USA; MusclePharm Sports Science Institute, MusclePharm Corp., Denver, CO USA; Maximum Mobile Fitness, Spearfish, SD USA

**Keywords:** Ergogenic aid, Hypertrophy, Safety, Elevatp, Purenergy

## Abstract

**Background:**

Increased ATP levels may enhance training-induced muscle accretion and fat loss, and caffeine is a known ergogenic aid. A novel supplement containing ancient peat and apple extracts has reported enhanced mitochondrial ATP production and it has been coupled with an extended-release caffeine. Therefore, the purpose of this investigation was to determine the effects of this supplement on body composition when used in conjunction with 12 weeks of resistance training.

**Methods:**

Twenty-one resistance-trained subjects (27.2 ± 5.6y; 173.5 ± 5.7 cm; 82.8 ± 12.0 kg) completed this study. Subjects supplemented daily with either 1 serving of the supplement (TRT), which consisted of 150 mg ancient peat and apple extracts, 180 mg blend of caffeine anhydrous and pterostilbene-bound caffeine, and 38 mg B vitamins, or an equal-volume, visually-identical placebo (PLA) 45 min prior to training or at the same time of day on rest days. Supervised resistance training consisted of 8 weeks of daily undulating periodized training followed by a 2-week overreach and a 2-week taper phase. Body composition was assessed using DEXA and ultrasound at weeks 0, 4, 8, 10, and 12. Vital signs and blood markers were assessed at weeks 0, 8, and 12.

**Results:**

Significant group x time (*p* < 0.05) interactions were present for cross-sectional area of the rectus femoris, which increased in TRT (+1.07 cm^2^) versus PLA (−0.08 cm^2^), as well as muscle thickness (TRT: +0.49 cm; PLA: +0.04 cm). A significant group x time (*p* < 0.05) interaction existed for creatinine (TRT: +0.00 mg/dL; PLA: +0.15 mg/dL) and estimated glomerular filtration rate (TRT: −0.70 mL/min/1.73; PLA: −14.6 mL/min/1.73), which remained within clinical ranges, but no other significant observations were observed.

**Conclusions:**

Supplementation with a combination of extended-release caffeine and ancient peat and apple extracts may enhance resistance training-induced skeletal muscle hypertrophy without adversely affecting blood chemistry.

## Background

Adenosine-5’-triphosphate (ATP) and ATP metabolites are involved in a myriad of biological processes including cardiac function, neurotransmission, blood flow, and muscle contraction [2, 26], and it is strongly suggested that increased ATP levels correlate with improved health and performance [18, 23, 40]. Direct supplementation with ATP seems to be effective for increasing ATP when measured in whole blood [23], but there have been mixed results [4, 11]. Therefore, an indirect approach for increasing ATP levels may be desirable. Previously, Reyes-Izquierdo and colleagues determined that a 150 mg dose of a blend of ancient peat and apple extracts significantly increased blood ATP compared to placebo in 18 [31] and 20 [32] subjects. In the latter research, blood ATP increased by 40 % at 60 min following ingestion, which dropped to 28 % at 120 min following ingestion. A muscle biopsy was conducted in one subject, and ATP levels were observed to increase in muscle tissue by 281 % at 60 min and 433 % at 120 min following ingestion [32]. Preliminary reports from this laboratory also support an increase in blood ATP levels, and suggest this occurs without an increase in reactive oxygen species, which may be associated with increased ATP production [12]. In fact, ancient peat and apple extracts may actually decrease reactive oxygen species [31], possibly blunting the increase caused by resistance training [3].

Previous reports on indirect ATP enhancement are sparse. However, the beneficial effects of ancient peat and apple extracts have recently been shown to positively enhance muscle mass [24]. Moreover, direct ATP supplementation may be efficacious for positively augmenting performance [23, 30, 44] and body composition [44]. Wilson et al. reported increases in whole-body lean mass as well as quadriceps muscle thickness in the ATP-supplemented group compared to placebo following a 12-week resistance training protocol.

There are a multitude of studies on the ergogenic properties of caffeine, which has been demonstrated to improve endurance [14], high-intensity sport [36], strength [8], and cognitive [27] performance. Moreover, caffeine may enhance body composition by way of increased metabolism. Caffeine has been demonstrated to increase fatty acid oxidation, energy expenditure, and thermogenesis [5, 17, 37], possibly via increased epinephrine, which can up-regulate both lipolysis and glycogenolysis, and is consistent with reports on caffeine supplementation and high-intensity exercise [21]. Astrup et al. has reported that caffeine supplementation increases vascular smooth muscle tone, another paralleled effect of epinephrine. These researchers also observed an increase in energy expenditure that correlated with plasma triglyceride levels [5]. Increased utilization of free fatty acids with caffeine supplementation has been reported in both overweight and normal weight individuals [1]. Furthermore, caffeine supplementation has been confirmed to reduce body weight and body fat in obese rats [39]. However, the chronic effects of caffeine supplementation on body fat in healthy humans are poorly understood.

While direct and indirect ATP supplementation may be capable of augmenting resistance training induced changes in body composition, minimal research exists on this topic. Even less is known when ancient peat and apple extracts are coupled with other ingredients. In addition, caffeine has been noted to increase metabolism as well as acute exercise performance. When used in conjunction with elevated ATP levels, it may be possible to simultaneously increase lean muscle mass, while reducing adiposity. Therefore, the primary purpose of this study was to assess the effects of a proprietary blend of ancient peat and apple extracts coupled with an extended-release caffeine on changes in body composition following 12 weeks of resistance training. The secondary purpose of this study was to assess the safety of the two ingredients. It was hypothesized that the supplement will aid increases in lean muscle mass and muscle hypertrophy and decrements in body fat without changing hematological safety markers.

## Methods

### Participants

Twenty-one healthy, resistance-trained, male subjects (27.2 ± 5.6y; 173.5 ± 5.7 cm; 82.8 ± 12.0 kg) completed the current investigation. Thirty-three subjects were recruited, and 3 subjects did not complete the study due to scheduling conflicts, 3 were not compliant with protocols, and 2 sustained injuries during the study unrelated to training or supplementation. Each subject was required to be capable of lifting 1.5 times their bodyweight in the squat and deadlift and 1 times bodyweight in the bench press. At baseline, the placebo (PLA) group was able to squat 1.71 ± 0.21, bench press 1.45 ± 0.19, and deadlift 2.17 ± 0.25 times their bodyweight, and the treatment (TRT) group was able to squat 1.58 ± 0.20, bench press 1.33 ± 0.20, and deadlift 1.97 ± 0.26 times their bodyweight. Approval for research with human subjects was obtained from the MusclePharm Sports Science Institute IRB, and subjects provided written informed consent documents prior to participation in the study.

### Experimental design

The precise methods of the present study have previously been published with the exception of a difference in treatment [24]. Subjects were randomly assigned to either the PLA (*n* = 11) or TRT (*n* = 10) groups. They were instructed to consume 1 serving (2 mL) of either PLA or TRT (150 mg ElevATP™, FutureCeuticals Inc., Momence, IL; 180 mg blend of caffeine anhydrous and PurEnergy™, Chromadex Inc., Irvine, CA; and 38 mg B vitamins) 45 min prior to training on training days or at a similar time of day on rest days. For a detailed composition of ElevATP™, composed of ancient peat and apple extracts, see [31]. The PurEnergy™ ingredient is composed of 43 % caffeine and 57 % pterostilbene. Total caffeine content per serving was ~129 mg. Supplement vials were weighed to ensure compliance. Subjects were resistance trained under the guidance of a certified strength and conditioning specialist 3 days per week for 8 weeks followed by a 2-week overreach and 2-week taper phase corresponding to weeks 9–10 and 11–12, respectively. A eucaloric diet consisting of 50 % calories from carbohydrates, 25 % from protein, and 25 % from fat was prescribed to all subjects at the onset of the study, and diets were tracked weekly via 3-day food logs. Total calories were determined for each individual based on the Mifflin St. Jeor equation adjusted for activity level. Subjects were measured at weeks 0, 4, 8, 10, and 12 for all body composition variables. Blood draws and vital sign measurements were conducted at weeks 0, 8, and 12. Body composition variables collected consisted of DEXA, which determined lean soft tissue (LST), fat mass (FM), and body fat percentage (% Fat), and ultrasound, which determined cross-sectional area (CSA), muscle thickness (MT), and fat thickness (FT).

### Resistance training program

Weeks 1–8 consisted of one muscle hypertrophy-oriented workout, one power workout, and one strength-oriented workout each week. The hypertrophy session consisted of barbell back squat, bench press, deadlift, incline bench press, hammer strength power squat machine, hammer strength isolateral bench press, leg press, leg extension, leg curl, and triceps extension performed for 3 sets of 6–12 repetition at 60-80 % 1RM intensity. The power session consisted of barbell back squat, bench press, and deadlift exercises performed for 5 sets of 2–5 repetitions with a goal of high velocity of movement with 40-60 % 1RM intensity. After performing the main exercises on the power day, subjects performed bent over row, pulldown, dumbbell row, shoulder press, lateral raise, and bicep curl exercises for the goal of muscle hypertrophy as described for chest and leg exercises. The strength session consisted of barbell back squat, bench press, deadlift, shoulder press, and pulldown exercises performed for 3 sets of 1–5 repetitions at 85–100 % 1RM intentisty. Following the resistance exercises on the strength day, participants performed 2–6 sets of 10–30s Wingates on a cycle ergometer with 2–4 min rest. Participants rested 48–72 h between each training day, and 30–120 s between sets on the hypertrophy day or 2-5 min between sets on the power and strength days. All exercises were completed within 60–120 min. During the overreach phase, participants performed high-volume workouts, similar to the hypertrophy-oriented workouts performed during weeks 1–8, on Monday through Thursday, with a strength-oriented workout or performance testing conducted on Friday for weeks 9 and 10, respectively. The taper weeks consisted of one power session on Mondays. On Wednesdays and Fridays, participants performed 1–3 heavy sets for 2–5 repetitions of the back squat, bench press, and deadlift, and each heavy exercise was immediately followed by the same exercise for 3 power-oriented sets, as previously described, before progressing to the next exercise.

### Measurements

Urine specific gravity was determined on each body composition testing day to ensure measurements were conducted in a euhydrated state. On 3 occasions, a participant was required to drink water until another urine sample could be submitted and verified for adequate hydration status. Body weight was determined using a calibrated column scale (SECA, Chino, CA). Body composition was analyzed for whole-body and segmental LST, FM, and % Fat using DEXA (Lunar Prodigy Primo, General Electric, Fairfield, CN) with enCORE software (Version 15, Madison, WN). Test-retest reliability for DEXA LST, % Fat, and FM, as measured using 15 subjects, resulted in an average ICC of >0.99. CSA, MT, and FT were determined using ultrasound (Logiq e, General Electric, Fairfield, CN). The minimum differences [43] needed to be considered a true change are 0.107 cm^2^ and 0.038 cm for CSA and MT, respectively. Ultrasonography determined CSA was measured at 75 % femur length, as defined as the distance from the anterior superior iliac spine to the superior aspect of the patella. MT of the quadriceps was measured at 50 % femur length, defined as the distance from the greater trochanter of the femur to the lateral epicondyle of the femur. MT was defined as the combined thickness of the vastus lateralis and vastus intermedius. The distance from the superficial aspect the femur to the deep aspect of the superficial fascia of the vastus lateralis was measured. FT was measured at the same site as MT, and it was defined as the distance from the superficial aspect of the vastus lateralis fascial layer to the deep aspect of the hypodermis. For MT, FT, and CSA, ICC was 0.99, 0.99, and 0.97, respectively. Vital signs were determined using an automated, digital sphygmomanometer (Omron Corporation, Kyoto, Japan). Blood draws were performed via venipuncture by a trained phlebotomist. Following a 10-h fast, all subjects submitted a blood sample for analysis in the morning to control for diurnal variations. Blood variables consisted of white blood cell count (WBC), red blood cell count (RBC), hemoglobin, hematocrit, mean corpuscular volume (MCV), mean corpuscular hemoglobin (MCH), mean corpuscular hemoglobin concentration (MCHC), red blood cell distribution width (RDW), platelets (absolute), neutrophils (percent and absolute), lymphocytes (percent and absolute), monocytes (percent and absolute), eosinophils (percent and absolute), basophils (percent and absolute), serum glucose, blood urea nitrogen (BUN), creatinine, estimated glomerular filtration rate (eGFR), BUN:creatinine, sodium, potassium, chloride, carbon dioxide, calcium, protein, albumin, globulin, albumin:globulin (A/G), bilirubin, alkaline phosphatase, aspartate aminotransferase (AST), alanine aminotransferase (ALT), total cholesterol, triglycerides, high density lipoprotein (HDL) cholesterol, very low density lipoprotein (VLDL), and low density lipoprotein (LDL) cholesterol. Blood variables were analyzed by a third party (Laboratory Corporation of America, Denver, CO). Inter-test reliability results from 12 men and women measured up to one week apart at the aforementioned laboratory resulted in no significant differences from day-to-day (*p* > 0.05) and an average inter-test Coefficient of Variation of 6.9 % for all tests.

### Statistical analyses

Repeated measures ANOVAs were performed to assess group, time, and group by time interactions with a significant p-value considered as ≤0.05. A Bonferroni post-hoc analysis was used to locate differences. Independent T-tests were conducted on the delta values for each time point. Dependent T-tests were conducted to determine within group differences for all body composition and hematology data with a significant interaction. Statistica (Version 10, Statsoft, Tulsa, OK) was used for all statistical analyses.

## Results

**Fig. 1:**
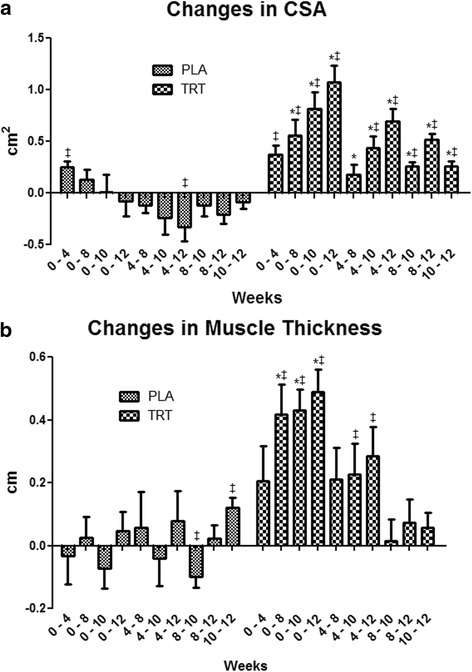
**a** Changes in CSA. Delta values between corresponding weeks are presented as mean ± standard deviation. * indicates significantly different from PLA. Significance was determined by Independent T-tests. ‡ indicates a significant (*p* < 0.05) within-group difference. **b** Changes in MT. Delta values between corresponding weeks are presented as mean ± standard deviation. * indicates significantly different from PLA. Significance was determined by Independent T-tests. ‡ indicates a significant (*p* < 0.05) within-group difference

**Fig. 2:**
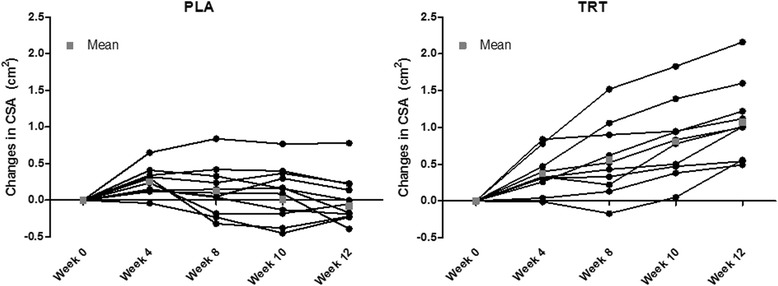
Individual Changes in CSA. Data presented represent the mean for each participant at all time points. The group mean is presented as a gray square at each time point

Significant time and group by time (*p* < 0.05) interactions were present for CSA (Fig. 1a, Fig. 2, and Table 1). CSA was greater in TRT versus PLA at weeks 8, 10, and 12. Moreover, CSA increased in TRT compared to PLA between all time points except for between weeks 0 and 4 using independent T-tests (*p* < 0.05). There were significant time and group by time (*p* < 0.05) interactions observed for MT (Fig. 1b, Table 1). MT increased to a greater extent in TRT than PLA from pre to weeks 8, 10, and 12. There was a significant group by time (*p* < 0.05) interaction for both left and right leg FM and % Fat, which decreased in TRT versus PLA (Table 2). Left and right leg % Fat did not reach significance in the post hoc analysis, yet independent T-tests conducted on the delta values revealed a significant difference between week 0 and weeks 4, 8, and 10 (Table 2). A significant main effect for time (*p* < 0.05) was found for body weight, LST, and LST of the arms, legs, and trunk, but no significant group by time interactions existed for these variables (Table 1). Moreover, a significant main effect for time (*p* < 0.05) was observed for FT, FM, and % Fat, yet no significant group by time interactions were observed for FM or % Fat of the arms or trunk (Table 2). No differences were observed for average daily calories, carbohydrates, fats, or proteins consumed each week throughout the study (*p* > 0.05).

**Table 1:** Lean Body Composition Data. Data are presented as mean ± standard deviation

Variable	Group	Pre	Week 4	Week 8	Week 10	Post	*p*
CSA (cm^2^)	PLA	3.60 ± 1.57	3.85 ± 1.49^a^	3.73 ± 1.32	3.61 ± 1.27	3.52 ± 1.39^bc^	<0.001
	TRT	3.75 ± 1.15	4.13 ± 1.14^a^	4.31 ± 1.16*^a^	4.56 ± 1.09*^abc^	4.82 ± 1.10*^abcd^	
MT (cm)	PLA	5.50 ± 0.72	5.47 ± 0.50	5.53 ± 0.74	5.43 ± 0.66^c^	5.55 ± 0.68^d^	<0.001
	TRT	5.33 ± 0.63	5.54 ± 0.73	5.75 ± 0.64*^a^	5.77 ± 0.61*^ab^	5.82 ± 0.54*^ab^	
LST (kg)	PLA	65.3 ± 8.1	65.2 ± 6.5	65.7 ± 6.6	66.2 ± 6.8	65.5 ± 6.9	0.62
	TRT	62.3 ± 8.5	63.3 ± 8.7	63.5 ± 8.7	64.1 ± 8.4	63.4 ± 8.5	
Body Weight (kg)	PLA	83.7 ± 10.5	86.3 ± 10.6	86.1 ± 11.5	87.3 ± 12.1	86.4 ± 11.7	1.00
	TRT	81.8 ± 14.0	84.0 ± 13.7	84.1 ± 15.0	84.9 ± 15.5	84.6 ± 16.4	
R Leg LST (kg)	PLA	10.6 ± 1.7	10.9 ± 1.9	10.9 ± 1.5	11.2 ± 1.7	10.9 ± 1.3	0.39
	TRT	10.3 ± 1.5	10.5 ± 1.6	10.6 ± 1.5	10.6 ± 1.6	10.8 ± 1.7	
L Leg LST (kg)	PLA	10.5 ± 1.5	10.8 ± 1.7	10.8 ± 1.4	11.1 ± 1.6	10.7 ± 1.1	0.35
	TRT	10.2 ± 1.4	10.6 ± 1.5	10.8 ± 1.6	10.7 ± 1.6	10.7 ± 1.7	
R Arm LST (kg)	PLA	4.7 ± 0.6	4.5 ± 0.5	4.3 ± 0.5	4.2 ± 0.5	4.1 ± 0.5	0.84
	TRT	4.4 ± 0.8	4.2 ± 0.7	3.9 ± 0.5	3.8 ± 0.5	3.7 ± 0.5	
L Arm LST (kg)	PLA	4.5 ± 0.5	4.5 ± 0.4	4.1 ± 0.5	4.0 ± 0.4	4.0 ± 0.5	0.61
	TRT	4.3 ± 0.7	4.1 ± 0.6	3.8 ± 0.5	3.7 ± 0.5	3.7 ± 0.4	
Trunk LST (kg)	PLA	30.8 ± 4.3	30.2 ± 2.9	31.2 ± 3.4	31.5 ± 3.5	31.6 ± 4.6	0.27
	TRT	29.2 ± 5.6	29.9 ± 4.9	30.3 ± 4.8	31.3 ± 4.6	30.5 ± 4.6	

*indicates significantly different from PLA at the corresponding time point. The p-value is derived from an ANOVA and representative of a main effect for group by time. Significant within-group time differences are indicated by ^a^(different from pre), ^b^(different from week 4), ^c^(different from week 8), and ^d^(different from week 10) for variables with a significant group x time interaction

**Table 2:** Adipose Body Composition Data. Data are presented as mean ± standard deviation

Variable	Group	Pre	Week 4	Week 8	Week 10	Post	*p*
FT (cm)	PLA	0.52 ± 0.20	0.49 ± 0.19	0.53 ± 0.19	0.48 ± 0.18	0.53 ± 0.16	0.96
	TRT	0.64 ± 0.43	0.63 ± 0.46	0.66 ± 0.43	0.62 ± 0.39	0.64 ± 0.43	
FM (kg)	PLA	16.7 ± 5.6	18.6 ± 6.3	18.4 ± 6.6	18.5 ± 6.9	18.5 ± 6.5	0.17
	TRT	17.4 ± 9.4	17.8 ± 9.3	18.3 ± 10.5	18.0 ± 10.4	18.8 ± 10.9	
% Fat	PLA	20.1 ± 5.4	21.8 ± 5.5	21.4 ± 5.8	21.3 ± 5.9	21.6 ± 5.7	0.14
	TRT	21.0 ± 8.8	21.2 ± 8.5	21.4 ± 9.4	20.9 ± 8.9	21.7 ± 9.3	
R Leg FM (kg)	PLA	2.5 ± 0.7	2.8 ± 0.9^a^	2.8 ± 0.9^a^	2.8 ± 0.9^a^	2.7 ± 0.8^a^	0.003
	TRT	2.8 ± 1.5	2.8 ± 1.4*	2.8 ± 1.5*	2.6 ± 1.3*	2.9 ± 1.5^d^	
L Leg FM (kg)	PLA	2.4 ± 0.7	2.8 ± 0.9^a^	2.7 ± 0.9^a^	2.7 ± 0.9^a^	2.7 ± 0.8^a^	0.01
	TRT	2.8 ± 1.5	2.8 ± 1.4*	2.8 ± 1.5*	2.7 ± 1.4*	2.9 ± 1.5^d^	
R Arm FM (kg)	PLA	0.66 ± 0.21	0.70 ± 0.22	0.61 ± 0.18	0.61 ± 0.20	0.61 ± 0.19	0.30
	TRT	0.72 ± 0.43	0.71 ± 0.39	0.62 ± 0.42	0.57 ± 0.34	0.59 ± 0.37	
L Arm FM (kg)	PLA	0.64 ± 0.21	0.70 ± 0.22	0.58 ± 0.18	0.59 ± 0.20	0.60 ± 0.19	0.18
	TRT	0.71 ± 0.42	0.71 ± 0.39	0.62 ± 0.42	0.56 ± 0.33	0.58 ± 0.37	
Trunk FM (kg)	PLA	9.9 ± 4.2	11.0 ± 4.2	11.2 ± 4.7	11.3 ± 4.8	11.4 ± 4.9	0.62
	TRT	9.8 ± 5.4	10.2 ± 5.6	10.8 ± 6.5	11.0 ± 7.0	11.3 ± 7.1	
R Leg % Fat	PLA	18.8 ± 4.2	20.2 ± 4.6^a^	19.9 ± 4.7	19.4 ± 4.5	19.7 ± 4.6	0.03
	TRT	20.6 ± 8.1	20.4 ± 7.8^†^	20.2 ± 8.2^†^	19.4 ± 7.5^†ab^	20.3 ± 7.7^d^	
L Leg % Fat	PLA	18.8 ± 4.2	20.2 ± 4.6^a^	19.9 ± 4.7	19.4 ± 4.6	19.7 ± 4.6	0.03
	TRT	20.6 ± 8.1	20.3 ± 7.8^†^	20.1 ± 8.2^†^	19.4 ± 7.5^†a^	20.3 ± 7.7^d^	
R Arm % Fat	PLA	12.3 ± 3.6	13.2 ± 3.3	12.3 ± 3.0	12.5 ± 3.5	12.9 ± 3.4	0.37
	TRT	14.1 ± 7.6	14.5 ± 7.7	13.6 ± 7.9	13.2 ± 7.6	13.5 ± 8.0	
L Arm % Fat	PLA	12.3 ± 3.6	13.3 ± 3.3	12.3 ± 2.9	12.5 ± 3.5	12.9 ± 3.4	0.35
	TRT	14.1 ± 7.7	14.6 ± 7.7	13.6 ± 7.9	13.1 ± 7.6	13.5 ± 8.0	
Trunk % Fat	PLA	23.8 ± 7.4	26.1 ± 7.5	25.5 ± 7.8	25.5 ± 8.0	25.5 ± 7.5	0.18
	TRT	24.1 ± 10.1	24.3 ± 9.6	24.9 ± 11.0	24.4 ± 10.4	25.2 ± 11.0	

*indicates significantly different from PLA at the corresponding time point. The p-value is derived from an ANOVA and representative of a main effect for group by time. † indicates significantly different from PLA at week 0 as determined by independent T-tests of the delta values. Significant within-group time differences are indicated by ^a^(different from pre), ^b^(different from week 4), ^c^(different from week 8), and ^d^(different from week 10) for variables with a significant group x time interaction

No changes were observed for systolic or diastolic blood pressure or heart rate. A significant group by time (*p* < 0.05) interaction was present for creatinine, which increased in PLA from pre to week 12 (TRT: 0.00; PLA: +0.15 mg/dL) and from week 8 to week 12 (TRT: −0.05; PLA: +0.09 mg/dL). There was a significant group by time (*p* < 0.05) interaction present for eGFR, which decreased in PLA from pre to week 12 (TRT: −0.70; PLA: −14.6 mL/min/1.73) and from week 8 to week 12 (TRT: +5.10; PLA: −7.73 mL/min/1.73). No other significant interactions were observed for any safety markers, and each marker remained within the physiological reference range (Tables 3, 4, and 5).

**Table 3:** Vital Signs and Blood Lipid Data

Variable	Treatment	PRE	Week 8	POST	Reference Interval	*p*
Systolic BP (mm Hg)	PLA	127 ± 12.0	126.0 ± 12.2	127.0 ± 12.2	90–120	0.93
	TRT	127.6 ± 12.8	127.7 ± 8.7	128.0 ± 10.4		
Diastolic BP (mm Hg)	PLA	76.1 ± 9.4	77.0 ± 8.0	76.4 ± 9.4	60–80	0.20
	TRT	76.6 ± 8.1	74.2 ± 9.4	78.2 ± 8.6		
Heart Rate (BPM)	PLA	68.9 ± 10.6	70.1 ± 10.1	70.0 ± 7.7	<100	0.43
	TRT	58.9 ± 12.0	62.1 ± 9.7	64.9 ± 9.4		
Total Cholesterol (mg/dL)	PLA	175.9 ± 41.9	175.1 ± 43.2	172.8 ± 41.0	100–199	0.39
	TRT	161.2 ± 19.4	173.1 ± 22.3	164.2 ± 29.3		
Triglycerides (mg/dL)	PLA	109.7 ± 53.0	81.4 ± 37.9	89.5 ± 33.3	0–149	0.055
	TRT	65.9 ± 22.2	74.5 ± 33.4	69.3 ± 36.5		
High Density Lipoprotein (mg/dL)	PLA	50.1 ± 13.3	47.6 ± 12.6	48.5 ± 11.2	>39	0.38
	TRT	53.8 ± 6.4	54.2 ± 10.2	50.8 ± 8.2		
Very Low Density Lipoprotein (mg/dL)	PLA	21.9 ± 10.5	16.3 ± 7.6	18.0 ± 6.8	5–40	0.06
	TRT	13.3 ± 4.5	15.0 ± 6.8	13.8 ± 7.3		
Low Density Lipoprotein (mg/dL)	PLA	103.9 ± 31.7	111.2 ± 31.2	106.4 ± 31.0	0–99	0.89
	TRT	94.1 ± 18.8	103.9 ± 19.9	99.7 ± 24.4		

Data are presented as mean ± standard deviation. The p-value is derived from an ANOVA and representative of a main effect for group by time

**Table 4:** Hematology Data

Variable	Treatment	PRE	Week 8	POST	Reference Interval	*p*
WBC (x10E3/uL)	PLA	5.7 ± 1.5	5.7 ± 1.0	5.7 ± 1.2	3.4–10.8	0.52
	TRT	5.9 ± 1.8	5.9 ± 0.7	6.4 ± 1.4		
RBC (x10E6/uL)	PLA	5.3 ± 0.3	5.4 ± 0.4	5.4 ± 0.3	4.14–5.80	0.17
	TRT	5.3 ± 0.3	5.4 ± 0.3	5.3 ± 0.2		
Hemoglobin (g/dL)	PLA	16.2 ± 1.2	16.4 ± 1.3	16.4 ± 1.0	12.6–17.7	0.15
	TRT	16.0 ± 1.7	16.0 ± 2.1	15.6 ± 1.8		
Hematocrit (%)	PLA	47.7 ± 3.0	48.5 ± 3.3	48.6 ± 2.7	37.5–51.0	0.31
	TRT	46.6 ± 3.6	47.5 ± 4.9	46.6 ± 4.3		
MCV (fL)	PLA	89.7 ± 3.1	89.7 ± 2.7	89.6 ± 2.7	79–97	0.54
	TRT	87.5 ± 7.0	87.9 ± 7.7	88.3 ± 8.7		
MCH (pg)	PLA	30.5 ± 1.1	30.3 ± 1.0	30.2 ± 0.9	26.6–33.0	0.77
	TRT	29.9 ± 3.2	29.6 ± 3.5	29.6 ± 3.5		
MCHC (g/dL)	PLA	34.0 ± 1.0	33.8 ± 0.6	33.7 ± 0.7	31.5–35.7	0.63
	TRT	34.2 ± 1.6	33.6 ± 1.6	33.5 ± 1.1		
RDW (%)	PLA	13.6 ± 0.5	13.4 ± 0.4	13.4 ± 0.4	12.3–15.4	0.06
	TRT	13.6 ± 1.2	13.9 ± 1.8	13.7 ± 1.7		
Platelets (x10E3/uL)	PLA	236.7 ± 29.1	239.4 ± 45.4	241.6 ± 31.6	155–379	0.61
	TRT	274.5 ± 61.0	286.4 ± 77.6	273.7 ± 79.5		
Neutrophils (%)	PLA	53.5 ± 8.3	49.0 ± 6.7	49.5 ± 9.1	40–74	0.23
	TRT	52.6 ± 12.2	50.4 ± 10.7	55.5 ± 10.9		
Lymphs (%)	PLA	35.1 ± 6.9	38.9 ± 6.5	38.2 ± 8.3	14–46	0.15
	TRT	34.3 ± 10.6	35.9 ± 9.0	30.8 ± 8.9		
Monocytes (%)	PLA	8.7 ± 1.8	9.4 ± 2.1	9.4 ± 2.4	4–12	0.77
	TRT	9.7 ± 2.0	10.0 ± 1.9	10.8 ± 3.6		
Eos (%)	PLA	2.1 ± 1.2	2.4 ± 2.4	2.5 ± 2.3	0–5	0.27
	TRT	2.8 ± 2.4	3.0 ± 2.4	2.3 ± 1.6		
Basos (%)	PLA	0.6 ± 0.7	0.4 ± 0.7	0.4 ± 0.7	0–3	0.43
	TRT	0.6 ± 0.7	0.7 ± 0.7	0.5 ± 0.7		
Neutrophils (Absolute) (x10E3/uL)	PLA	3.1 ± 1.4	2.8 ± 0.8	2.9 ± 1.0	1.4–7.0	0.41
	TRT	3.2 ± 1.6	3.0 ± 0.7	3.6 ± 1.4		
Lymphs (Absolute) (x10E3/uL)	PLA	1.9 ± 0.1	2.2 ± 0.3	2.1 ± 0.4	0.7–3.1	0.33
	TRT	1.9 ± 0.3	2.1 ± 0.5	1.9 ± 0.5		
Monocytes (Absolute) (x10E3/uL)	PLA	0.5 ± 0.2	0.5 ± 0.1	0.5 ± 0.2	0.1–0.9	0.52
	TRT	0.6 ± 0.2	0.6 ± 0.1	0.7 ± 0.2		
Eos (Absolute) (x10E3/uL)	PLA	0.1 ± 0.1	0.1 ± 0.1	0.1 ± 0.1	0.0–0.4	0.65
	TRT	0.2 ± 0.1	0.2 ± 0.1	0.2 ± 0.1		
Baso (Absolute) (x10E3/uL)	PLA	0.0 ± 0.0	0.0 ± 0.0	0.0 ± 0.0	0.0–0.2	0.10
	TRT	0.0 ± 0.1	0.0 ± 0.0	0.0 ± 0.0		

Data are presented as mean ± standard deviation. The p-value is derived from an ANOVA and representative of a main effect for group by time

**Table 5:** Blood Chemistry Data. Data are presented as mean ± standard deviation

Variable	Treatment	PRE	Week 8	POST	Reference Interval	*p*
Serum Glucose (mg/dL)	PLA	90.5 ± 11.0	89.3 ± 3.6	90.2 ± 5.4	65–99	0.84
	TRT	88.8 ± 6.1	89.6 ± 7.0	90.0 ± 6.8		
BUN (mg/dL)	PLA	17.3 ± 4.9	17.8 ± 3.9	17.5 ± 4.2	6–20	0.98
	TRT	15.1 ± 3.2	15.7 ± 3.1	15.7 ± 3.5		
Serum Creatinine (mg/dL)	PLA	0.97 ± 0.12	1.04 ± 0.12^a^	1.13 ± 0.17*^ab^	0.76–1.27	0.001
	TRT	1.05 ± 0.15	1.10 ± 0.11	1.05 ± 0.12*^‡^		
eGFR (mL/min/1.73)	PLA	105.6 ± 12.9	98.7 ± 12.5^a^	91.0 ± 14.9^a^	>59	0.01
	TRT	101.0 ± 15.8	95.2 ± 11.1	100.3 ± 12.9*^‡^		
BUN/Creatinine Ratio	PLA	18.0 ± 5.9	17.3 ± 4.0	15.8 ± 4.4	8–19	0.33
	TRT	14.4 ± 3.1	14.3 ± 1.8	15.0 ± 3.7		
Serum Sodium (mmol/L)	PLA	138.8 ± 1.8	139.6 ± 1.3	140.4 ± 2.1	134–144	0.43
	TRT	138.9 ± 1.7	138.7 ± 1.3	140.4 ± 1.3		
Serum Potassium (mmol/L)	PLA	4.4 ± 0.4	4.5 ± 0.5	4.3 ± 0.3	3.5–5.2	0.06
	TRT	4.4 ± 0.3	4.4 ± 0.3	4.6 ± 0.3		
Serum Chloride (mmol/L)	PLA	102.1 ± 1.6	101.0 ± 1.4	102.4 ± 2.2	97–108	0.92
	TRT	101.7 ± 1.8	100.7 ± 2.1	102.3 ± 1.6		
Carbon Dioxide (mmol/L)	PLA	23.6 ± 2.4	22.1 ± 1.5	22.5 ± 1.5	19–28	0.24
	TRT	23.1 ± 2.4	21.0 ± 1.2	22.8 ± 1.4		
Serum Calcium (mg/dL)	PLA	9.4 ± 0.4	9.4 ± 0.3	9.3 ± 0.3	8.7–10.2	0.45
	TRT	9.6 ± 0.4	9.5 ± 0.3	9.4 ± 0.4		
Serum Protein (g/dL)	PLA	7.1 ± 0.3	6.9 ± 0.3	6.9 ± 0.3	6.0–8.5	0.58
	TRT	7.2 ± 0.3	6.9 ± 0.2	6.9 ± 0.3		
Serum Albumin (g/dL)	PLA	4.4 ± 0.3	4.6 ± 0.2	4.6 ± 0.2	3.5–5.5	0.65
	TRT	4.5 ± 0.2	4.6 ± 0.2	4.6 ± 0.2		
Globulin (g/dL)	PLA	2.6 ± 0.2	2.3 ± 0.2	2.3 ± 0.2	1.5–4.5	0.83
	TRT	2.7 ± 0.2	2.3 ± 0.3	2.3 ± 0.3		
Albumin:Globulin Ratio	PLA	1.7 ± 0.2	2.0 ± 0.2	2.0 ± 0.3	1.1–2.5	0.76
	TRT	1.7 ± 0.2	2.0 ± 0.3	2.1 ± 0.3		
Bilirubin (mg/dl)	PLA	0.6 ± 0.2	0.6 ± 0.2	0.6 ± 0.2	0.0–1.2	0.06
	TRT	0.6 ± 0.3	0.7 ± 0.4	0.5 ± 0.2		
Alkaline Phosphatase (IU/L)	PLA	76.4 ± 13.8	81.3 ± 14.6	79.9 ± 16.1	39–117	0.53
	TRT	70.5 ± 20.8	79.5 ± 27.2	77.8 ± 23.4		
AST (IU/L)	PLA	25.5 ± 7.8	26.9 ± 7.4	25.8 ± 8.9	0–40	0.81
	TRT	25.1 ± 7.5	28.9 ± 11.6	27.2 ± 12.7		
ALT (IU/L)	PLA	23.7 ± 4.9	24.3 ± 7.0	22.5 ± 6.3	0–44	0.48
	TRT	23.5 ± 11.1	23.3 ± 8.6	26.5 ± 12.2		

*indicates significantly different from PLA at the corresponding time point. ‡ indicates significantly different from PLA at week 8. The p-value is derived from an ANOVA and representative of a main effect for group by time. Significant within-group time differences are indicated by ^a^(different from pre) and ^b^(different from week 8) for variables with a significant group x time interaction

## Discussion

The results of this study support the hypotheses. Although no interactions were observed for any DEXA-based measurements of LST between groups, both ultrasound-based measurements of CSA and MT increased while lower body measures of FM and % Fat decreased. No abnormal changes in vital signs or blood markers were detected. Creatinine and eGFR changed to a greater extent in PLA compared to TRT, indicating a normal variation in these markers. Furthermore, every blood marker remained within the accepted physiological range. With these considerations, it is unlikely that these changes were produced by supplementation.

Previous reports on direct and indirect ATP supplementation are in agreement with the present results. While there were no significant interactions reported for measures of body fat, Wilson et al. [44] observed significant increases in quadriceps muscle thickness in ATP-supplemented participants versus placebo following 12 weeks of periodized resistance training. There was also a significant increase in whole-body lean mass between groups over time, which only increased over time in the present study. Moreover, the present results concerning muscle mass are consistent with the observations of supplementation with ancient peat and apple extracts without caffeine [24]. Wilson et al. [44] reported no interactions for all measured blood markers, while the present study observed a possible effect for creatinine and eGFR, yet the relevance of these observations may be undue. This is also in agreement with Coolen and colleagues [13] who observed no changes in blood markers following 4 weeks direct supplementation with 5 g/day of ATP. Furthermore, multi-ingredient products containing caffeine have previously been reported to be safe for human consumption as determined by changes in hematology and hemodynamics, but these studies were of relatively short duration (2–4 weeks) compared to the present study [16, 42].

The blend of ancient peat and apple extracts may be capable of promoting skeletal muscle hypertrophy by increasing whole-blood ATP levels [31, 32] with a subsequent augmentation of blood flow. ATP and adenosine have been known to induce vasodilation following release from the erythrocytes via production of nitric oxide and prostacyclin [29, 38], and it has been recently demonstrated that exogenous ATP supplementation is capable of increasing exercise-induced blood flow [22]. Improved blood flow may increase nutrient delivery. Thus, there is a possibility for a greater effect of circulating amino acids [9, 28], glucose [6, 7], and oxygen [10], which may enhance anabolic signaling and/or acute exercise performance, leading to amplified chronic adaptations [10, 35].

The observed changes in FM and % Fat were likely due to the extended-release caffeine. While an extended-release caffeine is yet to be researched, studies have been conducted on caffeine anhydrous and naturally-occurring caffeine. Caffeine has previously been reported to increase fat oxidation in both lean [25] and overweight individuals [5]. Despite caffeine’s effects on metabolism, few studies have investigated the chronic effects of caffeine on body weight and body fat. Sugiura et al. [39] supplemented rats for 4 weeks with caffeine and found caffeine reduced intraperitoneal adipose tissue weights by over 50 % compared to control without a difference in food intake. However, long-term, placebo-controlled human clinical trials do not seem to confirm these results [41] without the addition of tea polyphenols [20], ephedra [41], or a combination of other ingredients [34]. It is also possible that caffeine had an effect on training. Duncan and Oxford have previously reported caffeine increases repetitions performed to failure while reducing perceptions of fatigue [15]. However, in the present study, there were no significant changes in volume performed between groups over time (data not presented). The present study cannot dismiss the potential for caffeine and a blend of ancient peat and apple extracts to have an effect similar to caffeine and tea polyphenols, as phenolic compounds are contained within the ancient peat and apple extracts ingredient blend, though in small amounts (<1 % efficacious dose) [31], and the combination of caffeine and tea polyphenols have previously been reported to produce reductions in body fat [19, 39]. Therefore, it may be the combination of caffeine and polyphenols rather than caffeine alone producing this effect, yet it is unlikely at this dose of polyphenols. Finally, the caffeine used in the present study has an extended-release effect due to its binding with pterostilbene, and pterostilbene has been previously reported to reduce BMI in overweight individuals [33].

The primary limitation of this study was the use of several effective ingredients simultaneously. The supplement studied was a multi-ingredient product containing ancient peat, apple extracts, B-vitamins, and an extended-release caffeine containing both caffeine anhydrous and pterostilbene, and while it is possible to speculate on the contributions of each of these ingredients, their individual contributions to the observed effects cannot be definitively interpreted. The present findings are thought to be due, at least in part, to the elevation of whole-blood or intramuscular ATP levels based on the results reported by Reyes-Izquierdo et al. on acute administration of the proprietary blend of ancient peat and apple extracts [31, 32]. However, this effect may not persist in a chronic setting, and the present study did not measure whole-blood or intramuscular ATP levels at any time point. The current study also did not feature a non-exercising control group, so the effects of the training program are unable to be determined.

## Conclusions

This is the first study to examine the effects of an ATP-enhancing supplement combined with an extended-release caffeine on body composition. There have been no previously published investigations regarding an extended-release caffeine, and there is a paucity of studies conducted on the long-term effects of caffeine on body composition without additional tea polyphenols. The combination of these ingredients appear to beneficially augment body composition when consumed during a periodized resistance training protocol. Increases in measures of muscle mass were likely produced by the blend of ancient peat and apple extracts, and the small reduction of body fat observed in the TRT group, versus an increase in the PLA group, is presumably due to the caffeine, although, a synergistic effect of these ingredients cannot be entirely dismissed from the present investigation. Athletes who benefit from increased muscle mass but not fat mass, such as bodybuilders and skill-position football players, as well as recreational athletes seeking improved body composition may benefit from the use of the present supplement combination. Future research should examine the extended-release caffeine used in the present study as a standalone supplement in conjunction with an exercise protocol designed for body fat reduction to determine its potential for reducing or maintaining body fat. Moreover, future research may be interested in exploring the effects of ancient peat and apple extracts in an endurance setting.

## Abbreviations

% Fat, percent fat; A/G, albumin to globulin ratio; ALT, alanine aminotransferase ; ANOVA, analysis of variance; AST, aspartate aminotransferase; ATP, adenosine triphosphate; BUN, blood urea nitrogen; CSA, cross-sectional area; DEXA, dual emissions x-ray absorptiometry; eGFR, estimated glomerular filtration rate; FM, fat mass; FT, fat thickness; HDL, high-density lipoprotein; LDL, low density lipoprotein; LST, lean soft tissue; MCH, mean corpuscular hemoglobin; MCHC, mean corpuscular hemoglobin concentration; MCV, mean corpuscular volume; MT, muscle thickness; PLA, placebo; RBC, red blood cell count; RDW, red blood cell distribution width; TRT, treatment; VLDL, very low density lipoprotein; WBC, white blood cell count
